# A threshold-free approach with age-dependency for estimating malaria seroprevalence

**DOI:** 10.1186/s12936-021-04022-4

**Published:** 2022-01-03

**Authors:** Irene Kyomuhangi, Emanuele Giorgi

**Affiliations:** CHICAS, Lancaster University, Sir John Fisher Drive, Lancaster, UK

**Keywords:** Malaria serology, Geostatistical model, Reversible catalytic model, Antibody acquisition model, Unified mechanistic model

## Abstract

**Background:**

In malaria serology analysis, the standard approach to obtain seroprevalence, i.e the proportion of seropositive individuals in a population, is based on a threshold which is used to classify individuals as seropositive or seronegative. The choice of this threshold is often arbitrary and is based on methods that ignore the age-dependency of the antibody distribution.

**Methods:**

Using cross-sectional antibody data from the Western Kenyan Highlands, this paper introduces a novel approach that has three main advantages over the current threshold-based approach: it avoids the use of thresholds; it accounts for the age dependency of malaria antibodies; and it allows us to propagate the uncertainty from the classification of individuals into seropositive and seronegative when estimating seroprevalence. The reversible catalytic model is used as an example for illustrating how to propagate this uncertainty into the parameter estimates of the model.

**Results:**

This paper finds that accounting for age-dependency leads to a better fit to the data than the standard approach which uses a single threshold across all ages. Additionally, the paper also finds that the proposed threshold-free approach is more robust against the selection of different age-groups when estimating seroprevalence.

**Conclusion:**

The novel threshold-free approach presented in this paper provides a statistically principled and more objective approach to estimating malaria seroprevalence. The introduced statistical framework also provides a means to compare results across studies which may use different age ranges for the estimation of seroprevalence.

**Supplementary Information:**

The online version contains supplementary material available at 10.1186/s12936-021-04022-4.

## Introduction

Thanks to increased diagnostic capacity, preventative measures and a scale-up of interventions, there has been an overall decrease in malaria burden worldwide [1, 2]. However, malaria still remains a significant global public health threat in sub-Saharan Africa, where *Plasmodium falciparum (P. falciparum)* is the predominant parasite. A total 229 million cases and 409,000 deaths have been estimated globally in 2019 [3]. Additionally, the decrease in malaria is heterogeneous across regions, countries and communities [2–6], posing additional challenges to malaria elimination efforts. These challenges require robust surveillance mechanisms which can adapt to the changing epidemiology, enabling a more targeted approach to intervention strategies [4, 7].

To estimate malaria exposure and transmission, analysis of human serology data has emerged as a viable alternative approach to disease risk metrics that are based on the detection of malaria parasites in humans and mosquito populations [8–10]. Because of the persistence of antibodies after infection, their concentration is less influenced by the seasonality of transmission and can be used as an indicator of the cumulative exposure to malaria. Additionally, antibodies, unlike the *Plasmodium* parasite, can be easily detected even in low transmission areas [8, 11–13].

Analysis of seroprevalence—i.e the proportion of ‘seropositive’ individuals—is often carried out using reversible catalytic models (RCM). These models allow for the estimation of seroconversion rates which quantify the transmission intensity and correspond to the rate at which individuals convert from seronegative to seropositive through exposure to malaria parasites over time [8, 9]. Alternatively, continuous antibody measurements can be used in antibody acquisition models to estimate boosting rates, another measure of transmission intensity, which represents the rate at which antibodies are boosted upon exposure to parasites [9, 10, 14]. Such indicators of transmission intensity can be used to inform decisions on intervention strategies by identifying hot-spots of transmission where individuals are likely to exceed a specified degree of exposure [15, 16].

To estimate seroprevalence, classification of individuals as seropositive or seronegative is required. The most commonly used approach is to identify a suitable threshold of antibody density beyond which individuals are classified as seropositive, and below as seronegative [8, 9, 11]. To this end, mixture distributions are first fitted to the antibody density data, assuming that continuous antibody measurements consists of two latent distributions, one for the seronegative and one for the seropositive populations. By using the point estimates of the mean, $\mu _{S^-}$ and standard deviation, $\sigma _{S-}$, of the seronegative distribution $S^-$, the seropositivity threshold is often set to $\mu +3 \sigma$ [9, 17–19], while other studies have instead used $\mu + 2 \sigma$ [20–22]. An alternative to this approach is to define thresholds based on the predictive probability of being seropositive resulting from the fitted mixture distribution [9].

The major drawback of threshold-based approaches is that the choice of the threshold is arbitrary and it is unclear to what extent this affects the results of the statistical analysis of serological data, as biased estimates of seroprevalence can in fact arise from the misclassification of individuals as seronegative or seropositive [23]. Additionally, in the case of the probability thresholds, individuals whose probability of belonging to either the seronegative or seropositive groups is close to 50% are often classified as ‘intermediate’, and are therefore excluded from analysis [9, 23]. Furthermore, the uncertainty around the estimated thresholds and probabilities used for the classification of individuals, is ignored.

In addition to these drawbacks, classical analysis of malaria serology data does not account for the age dependency of antibody distribution when calculating thresholds. Typically in mixture models, a threshold is obtained by assuming a constant mixing probability across all ages [14]. This assumption is questionable since, in malaria endemic settings, it is well known that antibody levels are in fact age-dependent [24, 25] and thus the likeihood of being seropositive is expected to increase with age. A 2011 study by Ster [26] incorporated age-dependency for varicella zoster virus serology mixture models, however, this principle has not been applied to malaria serology data

To address these issues, Kyomuhangi and Giorgi [14] proposed a unified modelling framework for the analysis of malaria serology data that uses the continuous antibody measurement rather than thresholds to estimate transmission parameters. However, as acknowledged by the authors, this modelling framework requires a larger amount of data than is usually available in serological studies to reliably estimate all the model parameters, thus limiting its applicability.

This paper proposes a novel modelling approach for the analysis of serological data that retains the same properties of the approach proposed in Kyomuhangi and Giorgi [14], but is also more parsimonious. More specifically, this novel approach satisfies the following requirements: (1) it accounts for age dependency of antibody levels; (2) it avoids the use of any threshold; and (3) it enables accounting for and propagating the uncertainty in the classification of seropositive and seronegative individuals. Using cross-sectional antibody data from Western Kenya, this paper demonstrates (1) the properties of this new methodology for estimating malaria seroprevalence, and (2) how to incorporate the uncertainty around the resulting seroprevalence estimates, using the standard RCM as an example. The discussion section in this paper explains how the principles used to develop this novel approach can be extended to more complex analysis of serological data.

## Methods

### Existing methods for estimating seroprevalence

This section outlines the most commonly used methods in the analysis of malaria serology data, to classify individuals as seropositive and seronegative, using a two-component mixture distribution.

Let $Y_{i}$ denote the log-transformed antibody measurement for the *i*-th individual in a sample, $S^-$ denote the seronegative classification, and $S^+$ denote the seropositive classification. Assuming independent and identically distributed realizations for a sample of *n* individuals, and $\mu$ to be the mean level of antibodies in the $S^-$ distribution, the density function of $Y = (Y_{1}, \ldots , Y_{n})$ is

1 $$\begin{aligned} f(y)=\prod _{i=1}^{n} \big [(1-p) f_{S^-}(y_i;\mu ,\sigma _{S^-}^2) + p f_{S^+}(y_i;\delta \mu ,\sigma _{S^+}^2) \big ] \end{aligned}$$

where $f_{S^-}$ is a univariate log-normal distribution with mean $\mu$ and variance $\sigma _{S^-}^2$ for the $S^-$ population, and analogously for $S^+$, with $\delta > 1$ being a multiplicative factor accounting for higher mean antibodies in the $S^+$ distribution. *p* is the probability of being $S^+$. Let $C_i$ and $C^*_i$ denote the random variables representing classification based on the mixture model and true classification of the *i*-th individual, respectively. Based on the seropositivity threshold $\kappa$, the classification of individuals, say $C_i$, into $S^+$ and $S^-$ is defined as

2 $$\begin{aligned} C_i= {\left\{ \begin{array}{ll} S^- &{} \text{ if } Y_{i} < \kappa \\ S^+ &{} \text{ if } Y_{i} \ge \kappa \end{array}\right. }. \end{aligned}$$

Since most statistical analyses of malaria serology data use $\kappa = \mu _{S^-} + 3 \sigma _{S^-}$ as the threshold, this will also be used in this paper.

### Proposed method for estimating seroprevalence

This paper proposes a novel modelling framework that overcomes the limits of the approach described in the previous section, by incorporating age-dependency into the mixture distribution in (1), and by propagating the uncertainty in the classification of individuals into $S^+$ and $S^-$ using a Monte Carlo approach.

In this framework, age dependency is introduced into (1) using linear regression, as described in Kyomuhangi and Giorgi [14]. Assuming $\mu (a_{i})$ to be the mean level of antibodies in the $S^-$ distribution for a given age $a_i$, (1) becomes

3 $$\begin{aligned} f(y) = \prod _{i=1}^n\left[ (1-p(a_i))f_{S^-}(y_i;\mu (a_{i}), \sigma _{S^-}^2) + p(a_i)f_{S^+}(y_i;\delta \mu (a_{i}), \sigma _{S^+}^2)\right] \end{aligned}$$

where $p(a_{i})$ is the probability of being $S^+$ at age *a*. Note that the seronegative distribution is also modelled as age-dependent to account for potential residual antibody levels due to previous infections. The age dependencies in *p*(*a*) and $\mu (a)$ are modeled using logit linear and log linear regression, respectively, such that

4 $$\begin{aligned} \log \left\{ \frac{p(a)}{1-p(a)}\right\} & = \alpha _1 + g_1(a) \nonumber \\ \log \{\mu (a)\} & = \alpha _2 + g_2(a) \end{aligned}$$

where $g_2(a)$ is a function of age that can be specified through empirical inspection of the data. In the case of $g_{1}(a)$, identifying a suitable specification may be more problematic because of the need to dichotomize the data. However, because it is well established that *p*(*a*) increases for increasing *a*, a pragmatic approach would be, for example, to specify a logit-linear regression on *a* as illustrated later in this paper. Note that predictor for these models can take other functional forms such as polynomials and smoothing splines to increase their flexibility.

Using the resulting mixture distribution, the predictive probability of belonging to the $S^+$ distribution for each sampled individual is computed by conditioning on the observed antibody measurement $Y_i=y_i$ and age $a_i$, to give

5 $$\begin{aligned} P(C^*_{i}=S^+ | y_{i},a_i) & = \frac{p(a_i)f_{S^+}(y_i;\theta _{S^+})}{(1-p(a_i))f_{S^-}(y_i;\theta _{S^-}) + p(a_i)f_{S^+}(y_i;\theta _{S^+})}\nonumber \\ P(C^*_{i}=S^- | y_{i},a_i) & = 1-P(C^*_{i}=S^+ | Y_{i}=y_{i},a_i) \end{aligned}$$

where $\theta _{S^-} = (\mu (a_{i}), \sigma _{S^-}^2)$ and $\theta _{S^+} = (\delta \mu (a_{i}), \sigma _{S^+}^2$). Based on the above expressions, when then simulate 10,000 classifications $C_i^*$ for a every single sampled individual. The resulting 10,000 data-sets generated from this process are then fed into the second stage of the analysis, which is explained in the next section.

There are two main advantages of this modelling approach. The first is that it avoids the use of a threshold $\kappa$ as in (2) and uses the generated samples $C_i$ to propagate the uncertainty of the classification into $S^+$ and $S^-$. The second is that the empirical approach used to account the age-dependency combines information across all ages as described in (4), and is therefore more efficient than fitting separate mixtures distribution for each age.

**Fig. 1:**
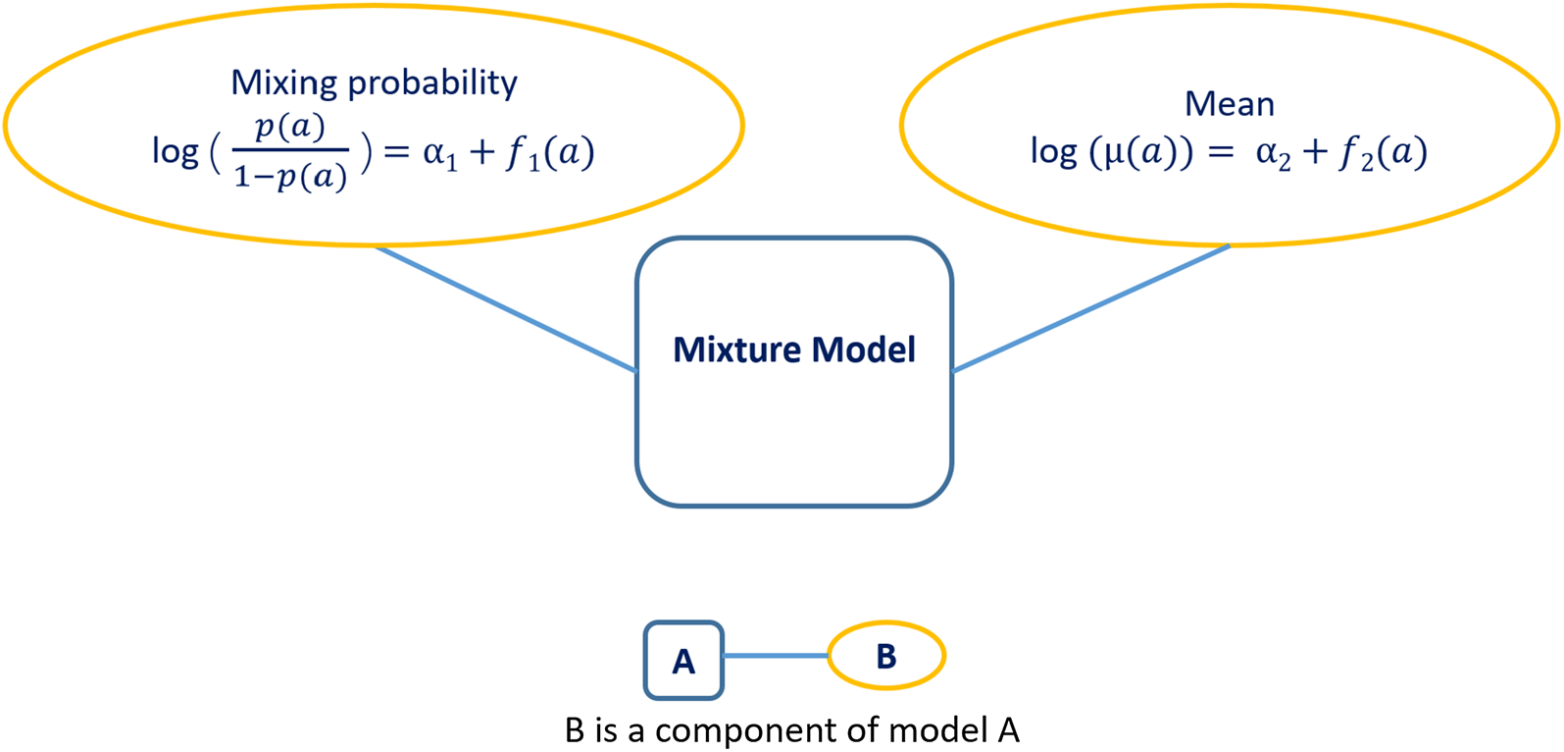
An illustration of the empirical model introduced in [14]. This model is used to describe the antibody mixture distribution as indicated in Eqs. (3) and (4)

The structure of this modeling framework is illustrated in Fig. 1.

### The reversible catalytic model

The RCM assumes that individuals are born $S^-$ and, after becoming $S^+$ upon exposure to malaria, can revert to $S^-$ in the absence of exposure. Since antibody data are believed to represent the cumulative exposure of individuals during their lifetime, an individual’s age prior to the sample collection is used as proxy for historical time.

Let $\lambda (a)$ denote the seroconversion rate for an individual at age *a* and $\omega$ the seroreversion rate. According to the RCM, the temporal dynamics that regulate the proportion of $S^+$ individuals of age *a*, i.e. *p*(*a*), are expressed by the following differential equation

6 $$\begin{aligned} \frac{dp}{da} = \lambda (a)(1-p(a))-\omega p(a). \end{aligned}$$

The seroconversion rate $\lambda (a)$ can be modelled using a variety of approaches, the simplest of which assumes constant transmission, i.e. $\lambda (a)=\lambda$ for all *a*. Due to poor identifiability of the seroreversion rate $\omega$, this is typically fixed and assumed to be constant across ages [9, 10, 14, 27]. Assuming a constant $\lambda$ and $\omega$ in (6) gives the following solution

7 $$\begin{aligned} p(a)=\frac{\lambda }{\lambda +\omega }\big (1-e^{-(\lambda +\omega )a}\big ). \end{aligned}$$

More flexible models could also be used to account for the temporal variation in $\lambda$, including a step-wise reduction or linear reduction in transmission [9, 27]. Additionally, other specifications of the RCM, for example the superinfection RCM [19] could be applied in the proposed approach. However in this paper, while comparing existing methods and the proposed approach described in the previous sections, attention is restricted to the RCM defined in the above equation for simplicity.

In order to propagate the uncertainty in classification of individuals as $S^+$ and $S^-$, for the purpose of estimating parameters of the RCM, the likelihood of a Binomial distribution with probability *p*(*a*) is maximized, as indicated in (7), for each of the 10,000 data-sets for the outcome $C_i$ as described in the previous sections. This gives 10,000 different estimates for $\lambda$, which is summarized by taking their mean and $2.5\%$ and $97.5\%$ quantiles.

The estimation of the model parameters is conducted using the maximum likelihood estimation method. Let $z_{i}$ denote the binary variable indicating seropositivity ($z_i=1$) or seronegativity ($z_i=0$) for the *i*-th individual; the likelihood function for the RCM in (7) is then

8 $$\begin{aligned} f({z_i}| {p(a_i)})=\prod _{i=1}^n p(a_i)^{z_i}(1-p(a_i))^{1-z_i} \end{aligned}$$

### Data

Data is taken from a cross-sectional survey which was conducted in Rachuonyo South District (34.75 to 34.95$^{\circ }$E, 0.41 to 0.52$^{\circ }$S), in the western Kenyan highlands (1400 m to 1600 m altitude), in 2011 over a 100 $\hbox {km}^2$ area. This survey was the baseline for a cluster-randomized controlled trial whose aim was to determine the community effect of interventions targeted at malaria prevalence hotspots. Further details of the study protocol can be found in [28]. At the time of the survey, malaria transmission in this area was described as low but highly heterogeneous, and seasonal, following peaks in rainfall, typically between March–June and October–November [16, 28].

The majority of malaria cases were attributed to *P. falciparum*, with *Anopheles gambiae sensu stricto (s.s.), Anopheles arabiensis,* and *Anopheles funestus* being the predominant vector species. Malaria control interventions at the time included distribution of insecticide-treated nets which had been ongoing for many years, and indoor residual spraying which started in 2009 [29, 30].

To generate the serology data, finger prick blood samples were collected from participants on filter paper and used to detect total Immunoglobulin G (IgG) antibodies against the blood-stage *P. falciparum* antigens, apical membrane antigen 1 (*Pf*AMA1) and merozoite surface protein-$1_{19}$ (*Pf*MSP$1_{19}$). Standard Enzyme-linked immunoassay (ELISA) methods [11, 31] were used to obtain Optical density (OD) values. Further details of the study design and data collection can be found in [28].

Analysis is first restricted to individuals between 1 and 16 years. Additional analysis on 1–20 year olds, 1–30 year olds, and 1–50 year olds is presented in Additional file 1. The data is split this way in order to investigate the effect of selecting different age-groups on the performance of M1 and M2. In what follows, the focus of analysis is the 1–16 year old age group.

**Fig. 2:**
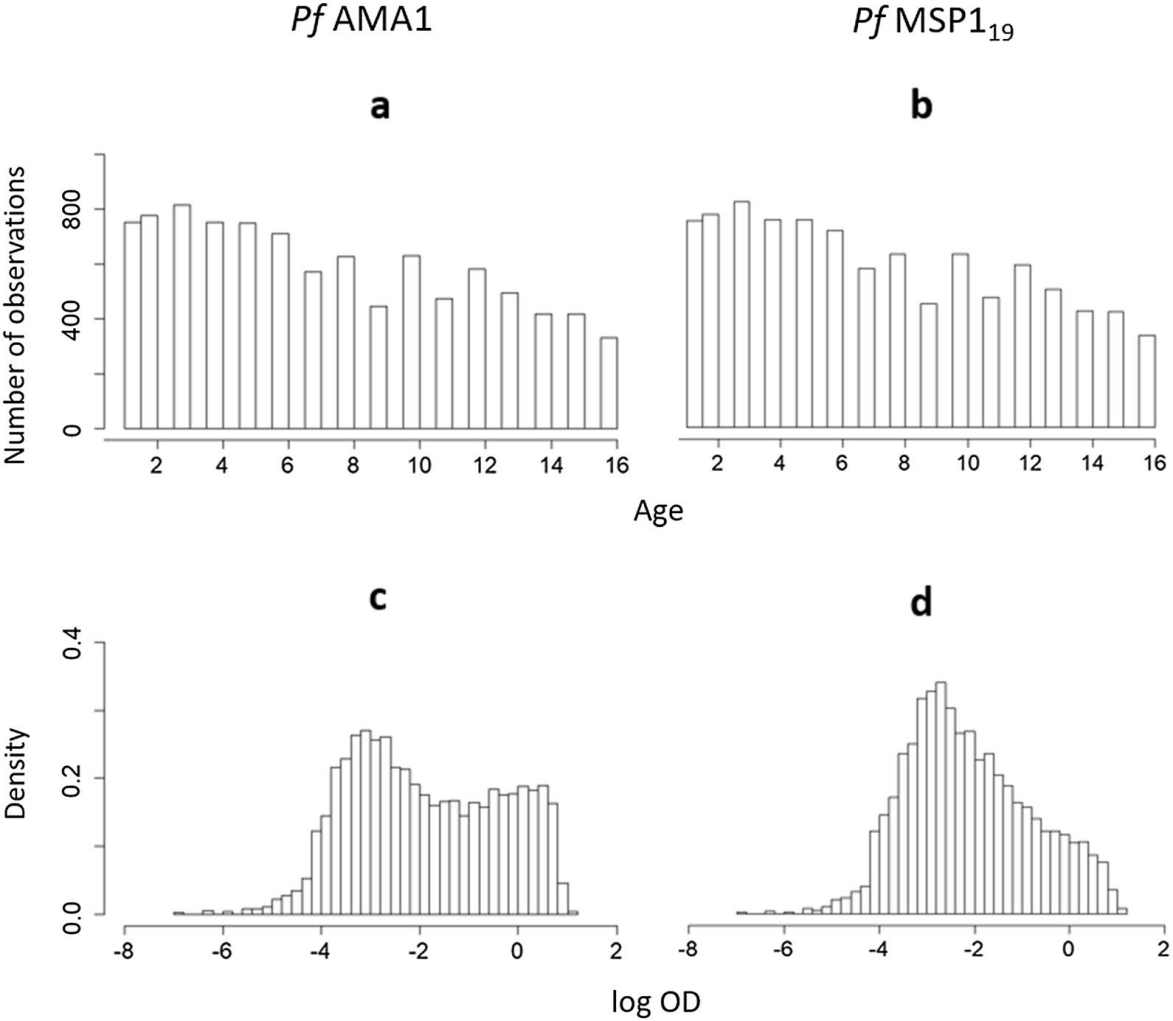
Descriptive plots of *Pf*AMA1 and *Pf*MSP$1_{19}$ antibodies for individuals between ages 1 and 16. The top row shows the age distribution, the bottom row shows the log OD distribution of individuals included in the analysis

The data-set consists of $n=9549$ children for the *Pf*AMA1 analysis and $n=9576$ for the *Pf*MSP$1_{19}$ analysis. Figure 2 shows the age and OD distributions of the individuals included in the analyses.

### Specifications of the model components

In this analysis, a comparison is conducted between two modelling approaches in the estimation of seroconversion rates, for both *Pf*AMA1 and *Pf*MSP$1_{19}$.

The first, which is denoted as M1, is the classic threshold-based approach as defined in (1), which considers seropositivity according to (2). After dichotomization of the antibody measurements, the RCM, as described by (6), is fitted using the maximum likelihood method.

**Fig. 3:**
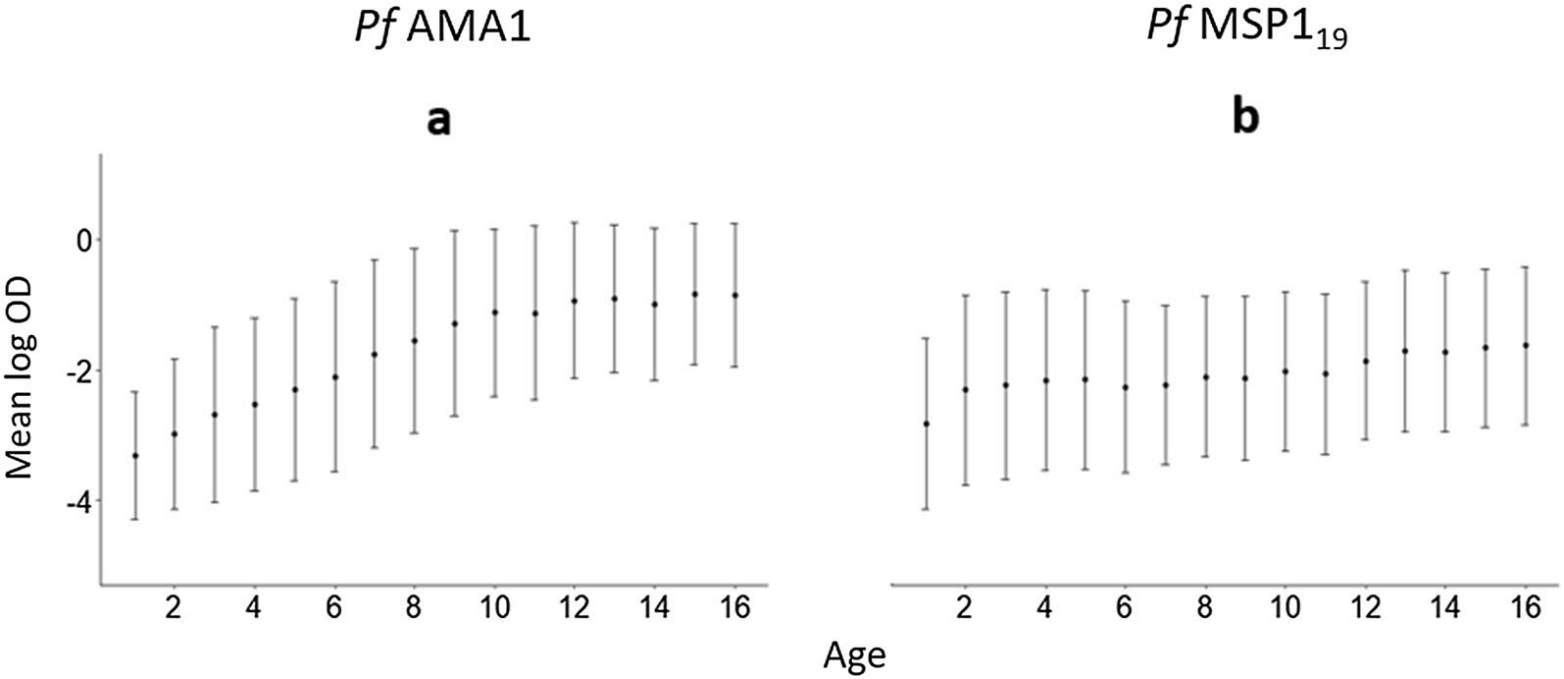
Exploratory analysis of *Pf*AMA1 and *Pf*MSP$1_{19}$ antibodies for individuals between ages 1 and 16. The figure shows the geometric mean OD by age, with associated error bars

The second modelling approach, which is denoted as M2, is the proposed threshold-free approach described in the previous sections. For this analysis, the age-dependency of the mixture models for the two antigens is modelled using an empirical approach. Based on the Fig. 3a for *Pf*AMA1, a linear spline with a knot at the age of 10 years is used. This is formally expressed as

9 $$\begin{aligned} \mu (a) = \exp \{\beta _0 + \beta _1 a + \beta _2 (a-10) I(a >10)\}, \end{aligned}$$

where $I(a >10)$ is an indicator function that takes value 1 if $a>10$, and 0 otherwise. For *Pf*MSP$1_{19}$, based on the trend observed in Fig. 3b, a log-linear model is used. This is given by

10 $$\begin{aligned} \mu (a) = \exp \{\beta _0 + \beta _1 a\}. \end{aligned}$$

To account for the age dependency in *p*(*a*), age is introduced as a logit-linear predictor of *p*(*a*), i.e.

11 $$\begin{aligned} p(a) = \frac{ \exp \{{\tilde{\beta }}_{0}+{\tilde{\beta }}_{1} a\}}{1 + \exp \{{\tilde{\beta }}_{0}+{\tilde{\beta }}_{1} a\}}. \end{aligned}$$

Note that M1 is recovered when all the regression parameters except $\beta _0$ and ${\tilde{\beta }}_{0}$ in (9), (10) and (11) are set to 0. Therefore for M1, only the estimates for $\beta _0$ and ${\tilde{\beta }}_{0}$ will be reported.

For both M1 and M2, due to the truncated nature of the antibody distributions, truncated log-normal distributions are used for both antigens. The upper limit, say $y_{max}(a_i)$, of the truncation is estimated for each age group as the maximum observed value of OD. Hence, the likelihood function in (3) now becomes

12 $$\begin{aligned} f(y) = \prod _{i=1}^n \frac{\left[ (1-p(a_i))f_{S^-}(y_i;\mu (a_{i}), \sigma _{S^-}^2) + p(a_i)f_{S^+}(y_i;\delta \mu (a_{i}), \sigma _{S^+}^2)\right] }{\left[ (1-p(a_i))F_{S^-}(y_{max};\mu (a_{i}), \sigma _{S^-}^2) + p(a_i)F_{S^+}(y_{max};\delta \mu (a_{i}), \sigma _{S^+}^2)\right] }, \end{aligned}$$

where $F_{S^+}$ and $F_{S^-}$ are the cumulative distribution functions of seropositive and seronegative probability distributions, respectively.

Finally, for the RCM, a range of values from 0.01 to 1 for $\omega$ are used, hence assuming that seroreversion events for individuals would occur between 1 and 100 years [8, 11, 15, 32]. Profile likelihood analysis indicated flat likelihood surfaces for *Pf*MSP$1_{19}$, and a tendency to $\omega =0$ for *Pf*AMA1 (see Additional file 1: Fig. S1), therefore $\omega$ is set to three values, namely 0.01, 0.5 and 1 to represent low, medium and high seroconversion rate respectively. In what follows, results are presented for the best performing value of $\omega$ for each antigen, i.e. $\omega = 0.01$ for *Pf*AMA1 and $\omega = 1$ for *Pf*MSP$1_{19}$. Note that these values are not the maximum likelihood estimates for $\omega$, but rather the best performing values out of the three choices stated above.

A summary of model parameters to estimate in this analysis is provided in Table 1. In order to compare how well M1 and M2 fit the data, the Akaike information criterion (AIC) is used. This is defined as $2p-2\log ({\hat{L}})$, where *p* is the number of parameters in the model and ${\hat{L}}$ is the value of the likelihood function evaluated at the maximum likelihood estimate. The AIC is used to quantify the goodness of fit of a model to the data while penalizing models that contain a larger number of parameters. The AIC can be used to compare models that are not nested, i.e. models that are not contained within each other. A lower AIC usually indicates a better fit to the data. All statistical analyses are conducted in the R version 4.1.1 (2021-08-10) [33] software environment, and maximization of the likelihood estimation is carried through unconstrained optimization using PORT routines as implemented in the “nlminb” function in R. The full reproducible code is available on GitHub (see ‘Availability of data and material’).

**Table 1:** Model specification for the analysis

Model	Equations	Age-dependency	Threshold	Parameters to estimate
M1	(1), (9), (10), (11), (7)	No	Yes	$\delta$, $\sigma _{S^-}^2$, $\sigma _{S^+}^2$, $\beta _0$, ${\tilde{\beta }}_{0}$, $\lambda$
M2	(3), (9), (10), (11), (7)	Yes	No	$\delta$, $\sigma _{S^-}^2$, $\sigma _{S^+}^2$, $\beta _{0}$, $\beta _{1}$, $\beta _{2}$, ${\tilde{\beta }}_{0}$, ${\tilde{\beta }}_{1}$, $\lambda$

## Results

**Fig. 4:**
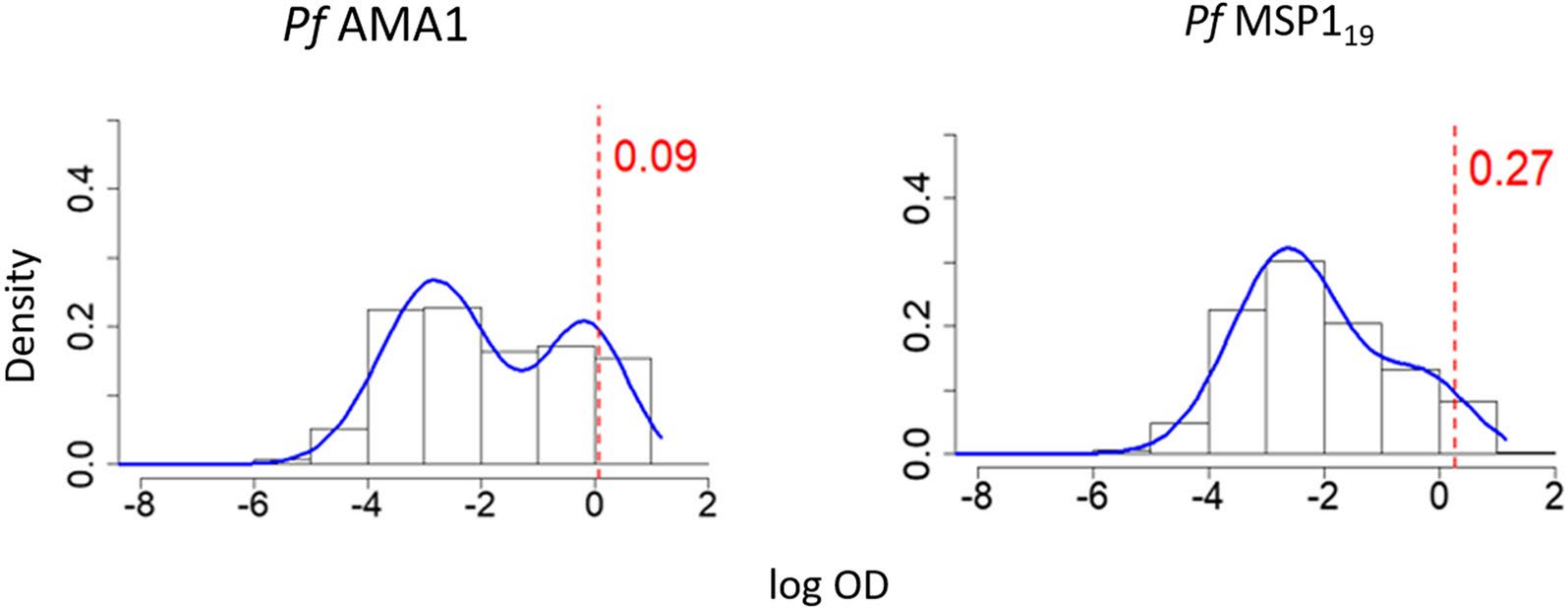
Mixture distributions of *Pf*AMA1 and *Pf*MSP$1_{19}$ antibodies for individuals between ages 1 and 16 using M1. These mixture distributions are derived from Eq. (1), and all the data of individuals aged 1–16 are analysed together. The red dotted lines illustrate the seropositivity thresholds ($\mu _{S^-} + 3 \sigma _{S^-}$), above which individuals are be classified as $S^+$ in traditional analysis

**Fig. 5:**
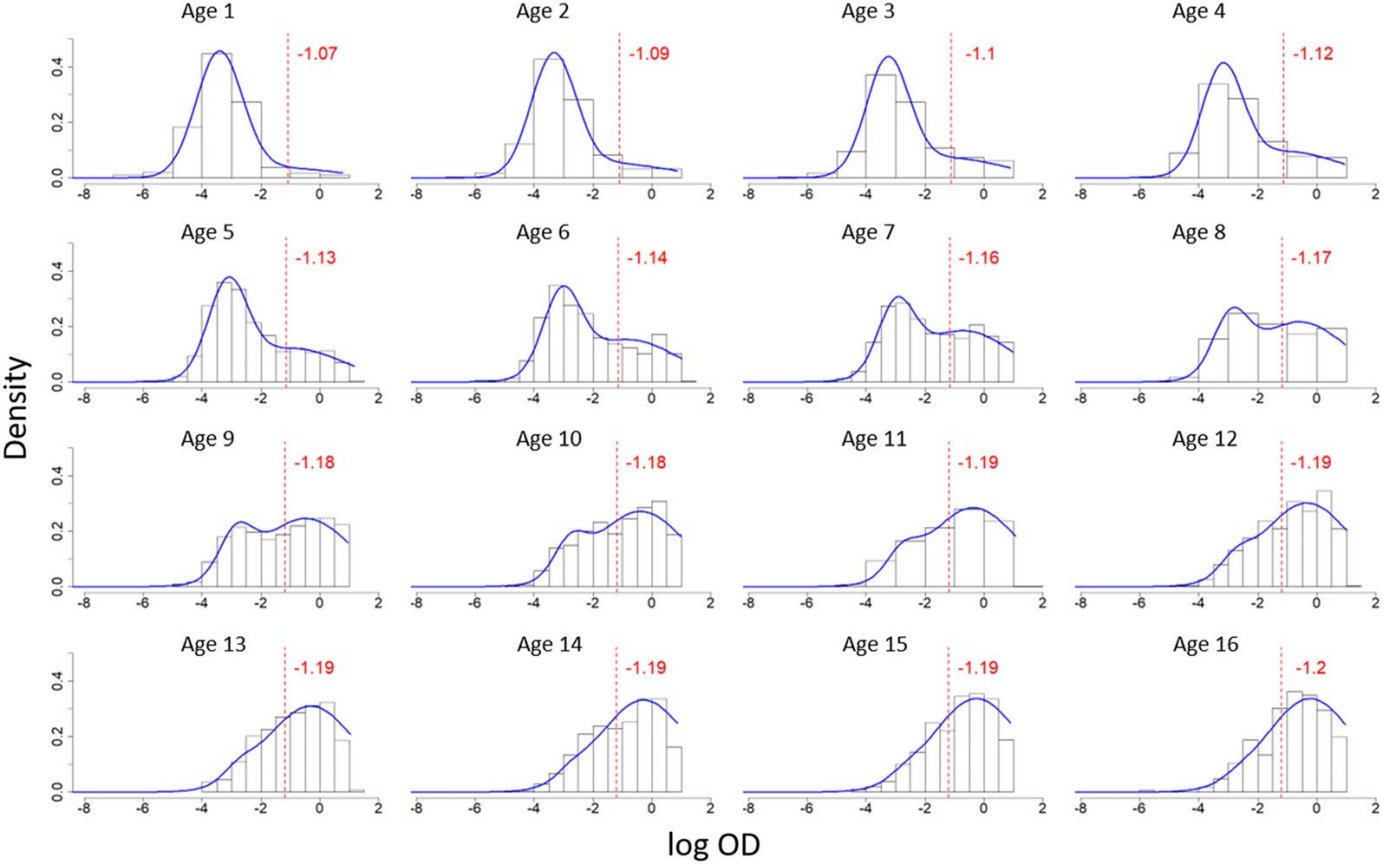
Age-dependent mixture distributions of *Pf*AMA1 antibodies for individuals between ages 1 and 16 using M2. The blue line shows fitted distributions derived from Eqs. (3), (9) and (11). The red dotted lines illustrate the seropositivity thresholds ($\mu _{S^-} + 3 \sigma _{S^-}$), above which individuals would be classified as $S^+$ in M1. Note that the red dotted lines are for illustration only—M2 does not use thresholds

**Fig. 6:**
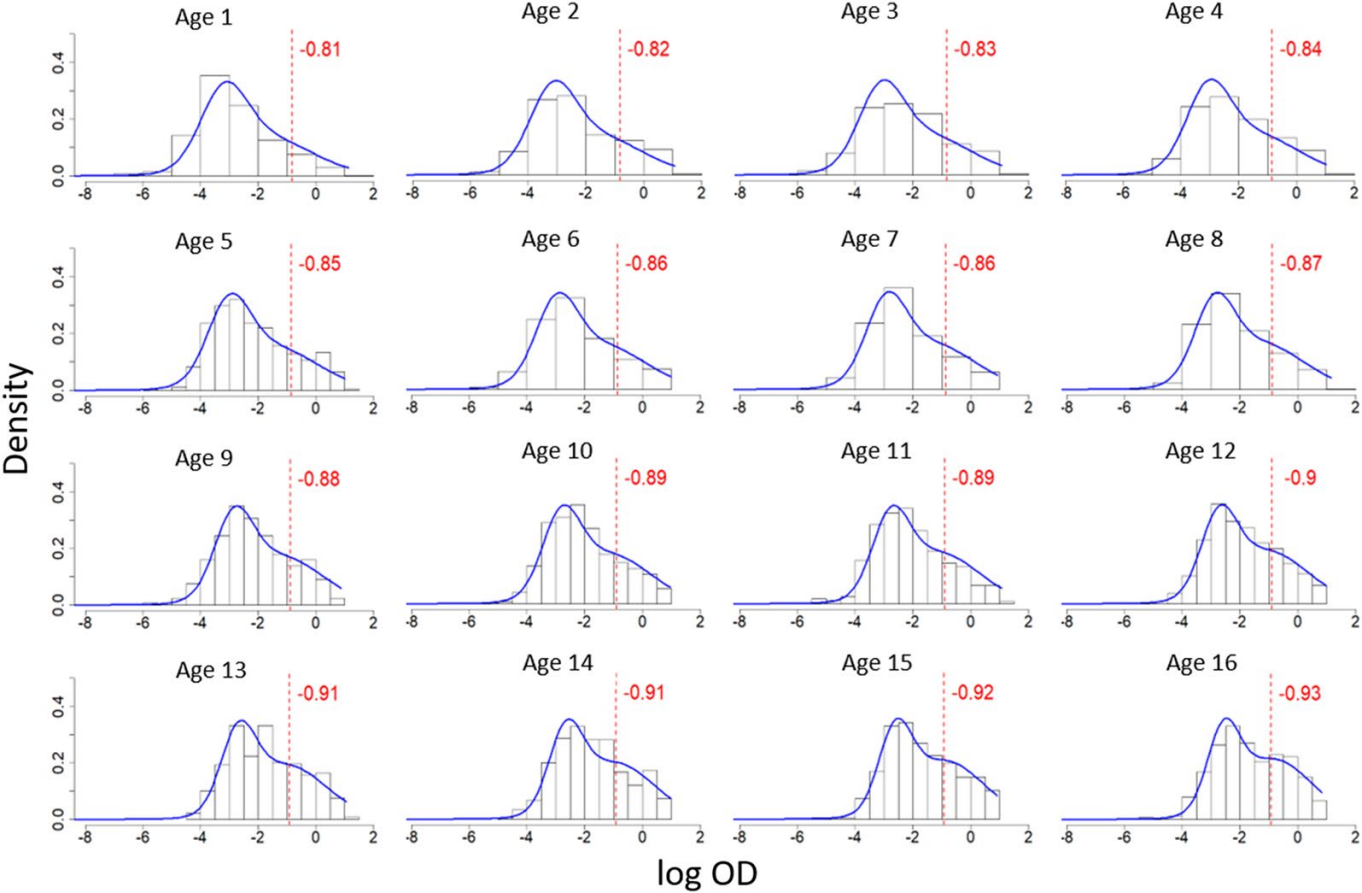
Age-dependent mixture distributions of *Pf*MSP$1_{19}$ antibodies for individuals between ages 1 and 16 using M2. The blue line shows fitted distributions derived from Eqs. (3), (10) and (11). The red dotted lines show the seropositivity thresholds ($\mu _{S^-} + 3 \sigma _{S^-}$), above which individuals would be classified as $S^+$ in M1. Note that the red dotted lines are for illustration only—M2 does not use thresholds

Figure 4 shows the antibody distribution and seropositivity thresholds for both antigens, as derived from M1. PfAMA1 shows greater separation between the components, as well as lower seropositivity threshold. A comparison of AIC in Table 2 shows a lower value for M2 than M1 for both antigens (29,669.940 versus 33,354.100 for *Pf*AMA1, and 31,162.920 versus 31,886.310 for *Pf*MSP$1_{19}$), indicating that the age-dependent mixture model in M2 is a better fit to the data compared to M1, which assumes a single mixture distribution across all ages. This age dependency is illustrated in Figs. 5 and 6, which show an increase in mean antibody levels and the mixture distribution with age. Of note, the increase is much more prominent for *Pf*AMA1, than for *Pf*MSP$1_{19}$.

**Table 2:** Maximum likelihood estimates with associated 95% CIs (within brackets) for M1 and M2, fitted to *Pf*AMA1 and *Pf*MSP$1_{19}$ antibody data. The Akaike Information Criterion (AIC) is also reported for the mixture models

		Parameter	M1	M2
*Pf*AMA1	Mixture model	$\beta _{0}$	− 2.338 (− 2.428, − 2.249)	− 3.164 (− 3.217, − 3.111)
		$\beta _{1}$		0.052 (0.045, 0.058)
		$\beta _{2}$		− 0.037 (− 0.052, − 0.023)
		${\tilde{\beta }}_{0}$	− 0.565 (− 0.671, − 0.460)	− 2.085 (− 2.281, − 1.890)
		${\tilde{\beta }}_{1}$		0.401 (0.371, 0.432)
		$\delta$	11.706 (10.778, 12.722)	30.613 (26.224, 35.764)
		$\sigma _{S^-}^2$	0.014 (0.011, 0.019)	$1.665\cdot 10^{-3}$ ($1.383\cdot 10^{-3}$, $2.003 \cdot 10^{-3}$)
		$\sigma _{S^+}^2$	0.884 (0.716, 1.092)	43.521 (25.898, 73.138)
		AIC	33354.100	29669.940
	RCM	$\lambda$	0.022 (0.020, 0.023)	0.175 (0.109, 0.286)
*Pf*MSP$1_{19}$	Mixture model	$\beta _{0}$	− 2.165 (− 2.2656, − 2.064)	− 2.915 (− 2.989, − 2.841)
		$\beta _{1}$		0.031 (0.028, 0.034)
		${\tilde{\beta }}_{0}$	− 1.220 (− 1.429, − 1.010)	0.081 (− 0.114, 0.277)
		${\tilde{\beta }}_{1}$		0.038 (0.022, 0.054)
		$\delta$	9.256 (8.624, 9.941)	11.698 (10.385, 13.193)
		$\sigma _{S^-}^2$	0.021 (0.015, 0.028)	$2.770\cdot 10^{-3}$ ($2.081\cdot 10^{-3}$, $3.687 \cdot 10^{-3}$)
		$\sigma _{S^+}^2$	0.994 (0.735, 1.346)	5.340 (3.387, 8.420) 31162.920
		AIC	31886.310	
	RCM	$\lambda$	0.060 (0.055, 0.066)	1.459 (0.760, 2.675)

Additionally, in both M1 and M2, the separation between the two components of the mixture distribution is more prominent in *Pf*AMA1 (Fig. 5) than in *Pf*MSP$1_{19}$ (Fig. 6) where there is poor separation of the $S^+$ and $S^-$ distributions. In the M2 *Pf*AMA1 analysis, the bi-modal distribution is more pronounced between the ages of 5 to 10 years, and less so in younger and older individuals. Figs. 5 and 6 also indicate that age modulates the seropositivity threshold.

**Fig. 7:**
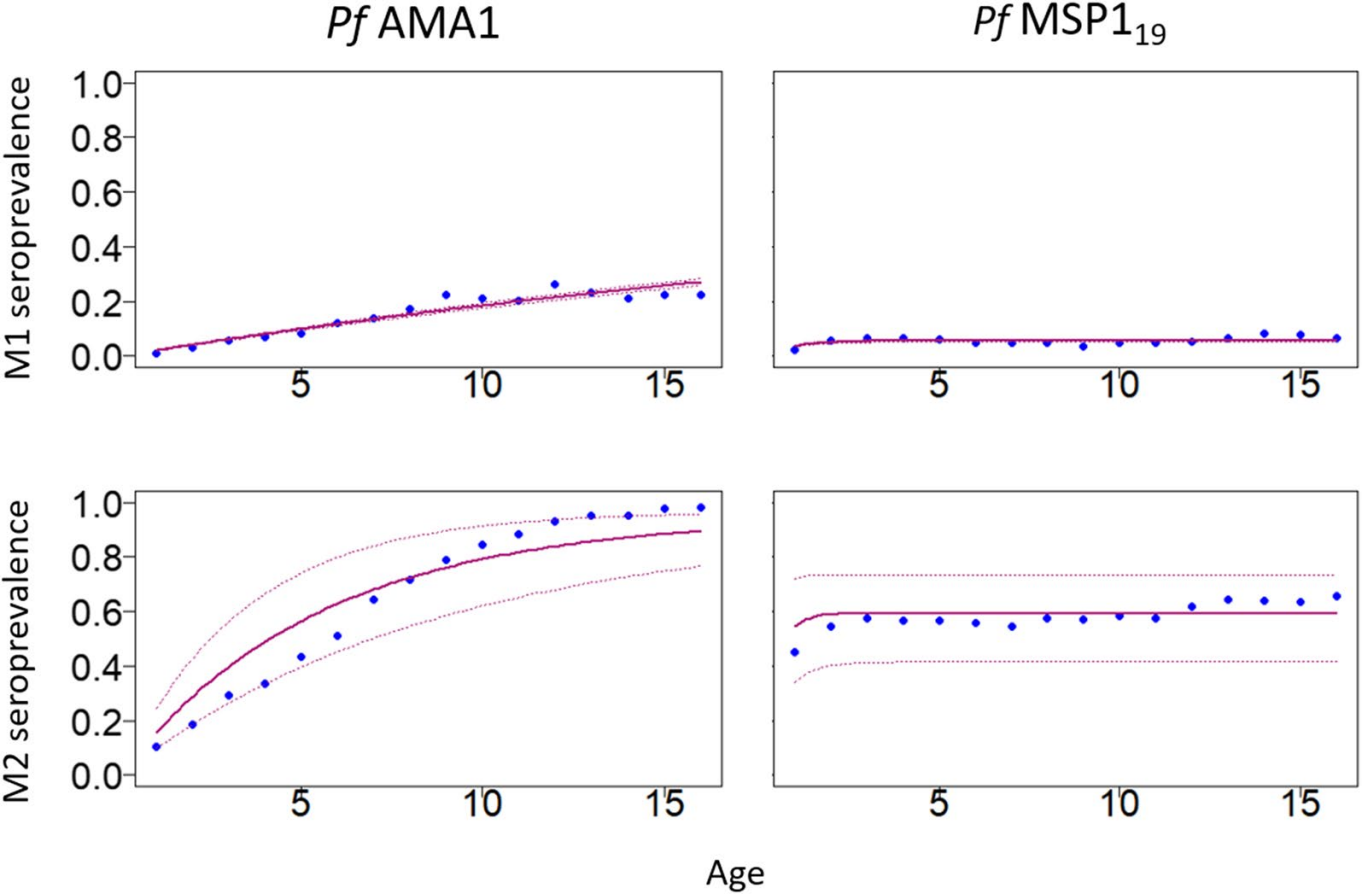
*Pf*AMA1 and *Pf*MSP$1_{19}$ seroprevalence estimates from M1, and seroprevalence distributions from M2, for individuals between ages 1 and 16. The top row shows M1 seroprevalence point estimates (blue dots), as well as the fitted seroprevalence curve (purple curve) and 95% CIs (purple dotted curves) from the RCM. The bottom row shows the mean of the seroprevalence distribution derived from M2 (blue dots), as well as the fitted seroprevalence curve (purple curve) and 95% CIs (purple dotted curves) from the RCM

Figure 7 shows the difference in seroprevalence estimation between M1 and M2, with overall higher estimates across age in the latter model. For both antigens, the uncertainty resulting from M2, as quantified by the 95$\%$ confidence intervals (CIs), in the seroprevalence estimates of the RCM is considerably larger than M1. This is because the M2 estimates are obtained by incorporating the uncertainty in the seropositivity classification, while M1 ignores this uncertainty, resulting in very narrow confidence intervals for M1.

Figure 7 also shows that the RCM fitted using M2, provides a good interpolation of the seroprevalence for *Pf*MSP$1_{19}$ but less so for the *Pf*AMA1. Although most of the seroprevalence points fall within the $95\%$ confidence interval, it is evident that, approaching 15 years of age, where the observed seroprevalence is not contained within the 95$\%$ intervals, the model underestimates seroprevalence. This is made more clear by visualizing the the y-axis of the plot in Fig. 7 on the logit-scale (see Additional file 1: Fig. S2). This indicates that, in the case of *Pf*AMA1, the assumptions of the standard RCM may not be fully supported by the data, which is undetected by the standard threshold-based model M1.

**Fig. 8:**
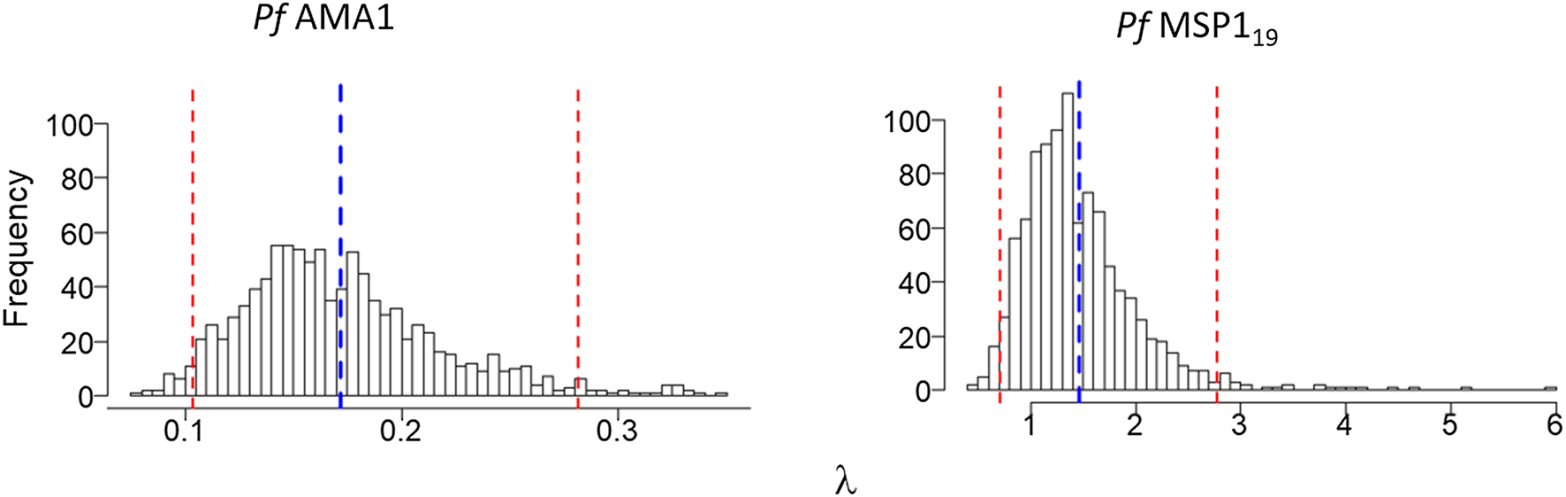
Distributions of the seroconversion rate $\lambda$ derived from M2 for *Pf*AMA1 and *Pf*MSP$1_{19}$. The mean and 95% CIs for $\lambda$ are indicated by blue and red dotted lines respectively. For *Pf*AMA1, these are 0.175 (0.109, 0.286), while for *Pf*MSP$1_{19}$, they are 1.459 (0.760, 2.675)

The distributions of $\lambda$ estimates derived from M2 for both antigens are shown in Fig. 8. For *Pf*AMA1, $\lambda$ is 0.175 (0.109, 0.286), while for *Pf*MSP$1_{19}$, this is 1.459 (0.760, 2.675). Note that these estimates represent the mean, 2.5% and 97.5% quantiles from the Monte Carlo distributions of the maximum likelihood estimates for $\lambda$.

Finally, Additional file 1: Figs. S3 and S4 show that M2 is consistent in the estimation of both seroprevalence and $\lambda$, even when different age groups are considered in analysis, unlike M1. Additional file 1: Fig. S5 also shows the additional variation in seroprevalence estimates for M1 when different seropositivity thresholds are used. Note the marked decrease in seroprevalence estimates as the threshold increases (see Additional file 1).

## Discussion

This paper presents a threshold-free method for estimating seroprevalence that incorporates the age dependency of malaria antibodies in the classification of individuals into seropositive and seronegative. Additionally, the paper demonstrates how the uncertainty of this classification can be accounted for in a the RCM. Note that this approach can be applied to other types of analyses that require the use of models different from the RCM. For example, if the goal of the study is to map seroprevalence data within a study area, the simulated classifications (previously denoted by $C_i$) could be used as the input of a geostatistical model whose results are then summarized in a similar fashion as presented for the RCM in this paper.

In the application of the proposed modelling framework to the RCM, seroprevalence is modelled into two different stages, using two different approaches: first, in a mixture distribution, using a logit-linear regression; and secondly, in an RCM, using Eq. (7). This raises the question of a mathematical inconsistency since both equations cannot be simultaneously true. Note that this issue also applies to previous work which uses threshold-based RCMs [8, 11, 15, 17], whereby the threshold is first generated using a constant mixing probability, which would correspond to an intercept-only logit-linear regression in this paper, and is then modelled using Eq. (7). To avoid this issue, one solution would be to replace the logit-linear regression on age for seroprevalence, with Eq. (7), hence embedding the assumptions of the RCM directly into the mixture distribution. However, the preference remains with the approach illustrated in this paper for the following reasons. First, the use of a logit-linear regression on age in the mixture distributions allows us to develop an empirical approach that is more flexible than an RCM and can better capture the variations of the antibody distributions across age. Secondly, the use of the RCM-based Eq. (7) for seroprevalence also in the mixture distributions would yield a circular argument, whereby the outcome to be modelled with the RCM would be already generated under an RCM, thus making any validation of the RCM assumptions a vain exercise. As shown in the case-study with western Kenya data, the approach presented in this paper can in fact better detect the inadequacy of the RCM than the current threshold-based approach.

The results in this paper show clear age-dependency in the mean antibody levels, the mixture distribution, and the threshold. The differences between *Pf*AMA1 and *Pf*MSP$1_{19}$ indicate that the magnitude of this dependency is likely dependent on the type of antigen and the dynamics of the immune response to it. Notably, results provide evidence that different combinations of age-groups in analysis lead to different seropositivity thresholds and, therefore different seroprevalence estimates. This inconsistency has significant implications for control programmes which rely on these results to direct intervention strategies. A key advantage of the threshold-free approach is that it is unaffected by the age limits considered for the analysis.

Furthermore, different definitions of the seropositivity threshold (i.e. between 2 and 5 standard deviations of the mean of the seronegative distribution) are an additional source of inconsistency in current literature. This makes the comparability of results reported across malaria serology studies more difficult. Avoiding the use of an arbitrary threshold, as described in this paper, provides a statistically rigorous solution to this problem and facilitates the comparison of results across studies.

The limitations of dichotomizing continuous measurements into positive and negative for statistical analysis are well established in the literature, and include loss of information which affects the ability to reliably recover regression relationships, as well as reducing the the precision of parameter estimates [34–36]. However when the scientific interest is in estimating seroprevalence—as this paper sets out to do—rather than modeling the dynamics that affect mean antibody antibody levels, dichotomization may be appropriate. This is because the approach presented in this paper results in a more parsimonious model than the unified mechanistic model presented in Kyomuhangi and Giorgi [14], allowing for a more efficient estimation of parameters that only modulate seroprevalence.

Depending on the degree of overlap between the seronegative and seropositive populations in the sample, mixture models can be difficult to estimate. The *Pf*MSP$1_{19}$ analysis illustrates this key limitation. Due to the poor separation of the seronegative and seropositive populations, the estimate for $\lambda$ shows a large value, which is inconsistent with other epidemiological data from the study site. This poor separation could be a biological feature of the antibody response to *Pf*MSP$1_{19}$, or due to poor dynamic range of the serological assay that generated the data. Similarly, in areas of high transmission where the majority of the population is seropositive [10, 13], or in elimination settings where there are very few seropositive cases, estimating the model parameters may be difficult. In these scenarios, if prior knowledge on some of the components of the model is available, Bayesian methods of inference can be used to alleviate estimation issues though the specification of suitable prior distributions. Additionally, to deal with skewness of the antibody distributions which can still persists after taking the logarithmic transformation, a mixture of skew-Normal distributions can be used in the mixture model to model the left asymmetry of the seropositive population [37].

When fitting the RCM, the seroreversion rate may also be difficult to estimate, hence $\omega$ is usually fixed [9]. In this paper, the simplest form of the RCM, which assumes constant transmission was used. This ignores possible changes in transmission due to, for example interventions in the recent past. While the resulting seroprevalence curves from the RCM do not fit the data very well in Fig. 7, the majority of seroprevalence points fall within the 95% CIs of the seroprevalence curves. Several studies have proposed modifications which relax this assumption of constant transmission [9, 17, 27, 38], and each of these can be fitted by using the Monte Carlo approach proposed in this paper to propagate the uncertainty in the classification of seropositive individuals.

## Conclusion

This paper proposes a new threshold-free method for estimating malaria seroprevalence which accounts for age dependency of antibodies through regression, and incorporates uncertainty around the estimates in subsequent analysis of the data. This method is more robust to varying conditions of analysis and provides more consistent estimates than the traditional threshold-based approach.

## Supplementary Information

**Additional file 1.** Additional figures.

## Data Availability

The dataset included in this paper is not publicly available but may be requested from Prof Chris Drakeley at The London School of Hygiene and Tropical Medicine. The R script to run both M1 and M2 is available from the authors upon request, and accessible on Github (https://github.com/kyomuhai/Kyomuhangi-and-Giorgi_-thresholdfree). Supplementary material is available as part of this submission.
