# Supplementary material for: A threshold-free approach with age-dependency for estimating malaria seroprevalence
Source: Malar J. 2022 Jan 3;21:1. doi: 10.1186/s12936-021-04022-4 (PMC8725324; doi:10.1186/s12936-021-04022-4)
Supplement: Supplementary file 1 — Additional file 1. Additional figures. [file 12936_2021_4022_MOESM1_ESM.pdf]

A threshold-free approach with age-dependency for estimating malaria seroprevalence Supplementary material.

Irene Kyomuhangi *[1] and Emanuele Giorgi[1]

[1]CHICAS, Lancaster Medical School, Lancaster University, UK
*i.kyomuhangi@lancaster.ac.uk

A Profile likelihood for different values of ω

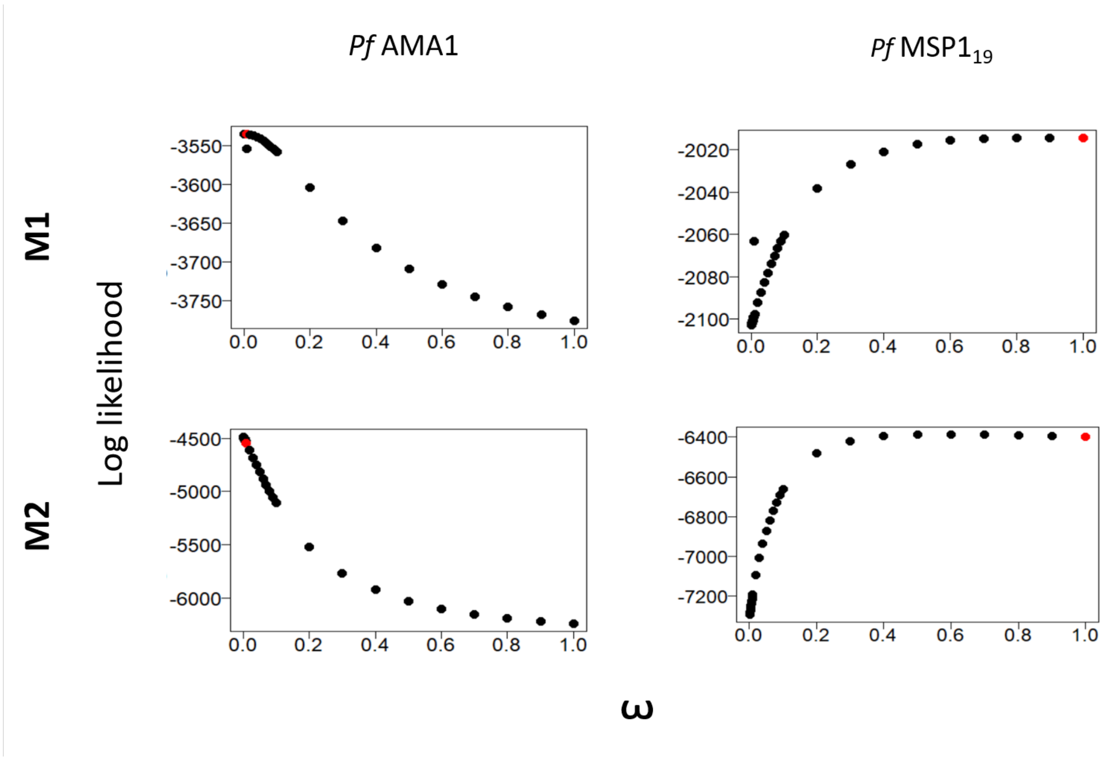

Figure. S 1: Profile likelihood analysis for different values of ω in the *Pf* AMA1 and *Pf* MSP1_{19} analyses. Note that for M1, the dots indicate the maximized log-likelihood function for one data-set (i.e a point estimate) while for M2, the dots indicate the mean of the maximized log-likelihood function for 10,00 data-sets (i.e. the mean of a distribution). Red dots show values of ω selected for each antigen, i,e ω=0.01 for *Pf* AMA1 and ω=1 for *Pf* MSP1_{19}

*Corresponding author

B Logit-transformed prevalence estimates from M2

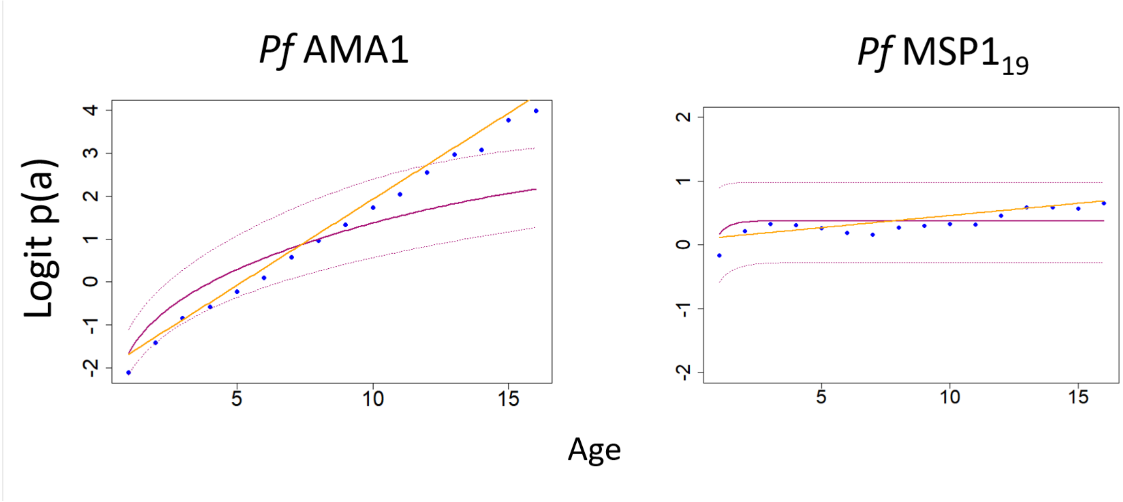

Figure. S 2: Logit-transformed prevalence estimates from M2. The mean of the seroprevalence distribution is indicated by blue dots; the purple solid and dotted curves represent the fitted seroprevalence and 95% CIs, respectively, from the RCM; and the orange line indicates the fitted seroprevalence estimate from the age-dependent mixture model, as defined by equation (11).

C M1 and M2 analysis using data from different age groups

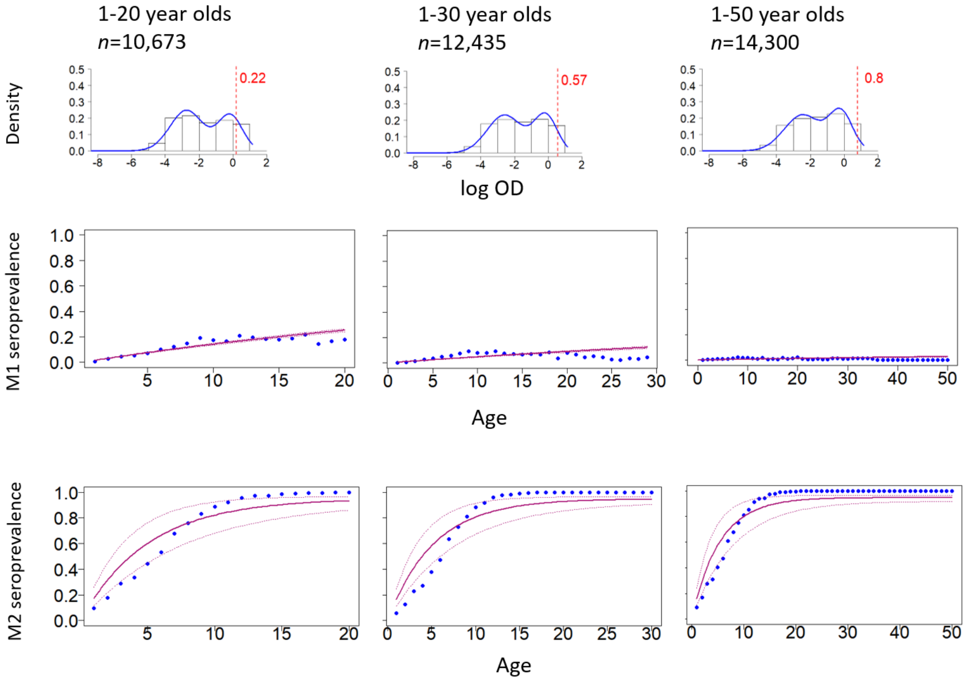

Figure. S 3: Analysis of *Pf*AMA1 mixture distributions using different age-groups in both M1 and M2. The mixture distributions in the top row are derived from M1 (see equation (1)), and show the seropositivity thresholds (red dotted lines represent $\mu_{S-} + 3\sigma_{S-}$) when different age groups are used in analysis. The middle row shows M1 seroprevalence point estimates (blue dots), as well as the fitted seroprevalence curve (purple curve) and 95% CIs (purple dotted curves) from the RCM. The bottom row shows the mean of the seroprevalence distribution derived from M2 (blue dots), as well as the fitted seroprevalence curve (purple curve) and 95% CIs (purple dotted curves) from the RCM.

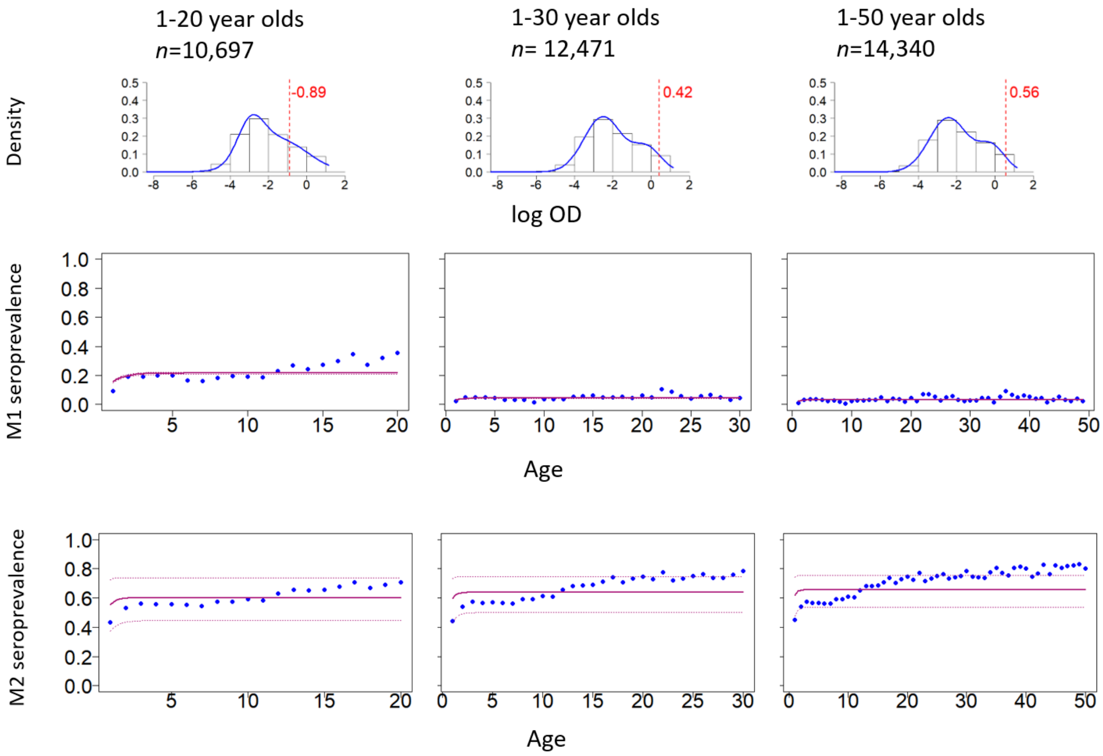

Figure. S 4: Analysis of *Pf*MSP1$_{19}$ mixture distributions using different age-groups in both M1 and M2. The mixture distributions in the top row are derived from M1 (see equation (1)), and show the seropositivity thresholds (red dotted lines represent $\mu_{S-} + 3\sigma_{S-}$ thresholds) when different age groups are used in analysis. The middle row shows M1 seroprevalence estimates (blue dots), as well as the fitted seroprevalence curve (purple curve) and 95% CIs (purple dotted curves) from the RCM. The bottom row shows the mean of the seroprevalence distribution derived from M2 (blue dots), as well as the fitted seroprevalence curve (purple curve) and 95% CIs (purple dotted curves) from the RCM.

D M1 analysis using different seropositivity thresholds for individuals aged 1-16 years

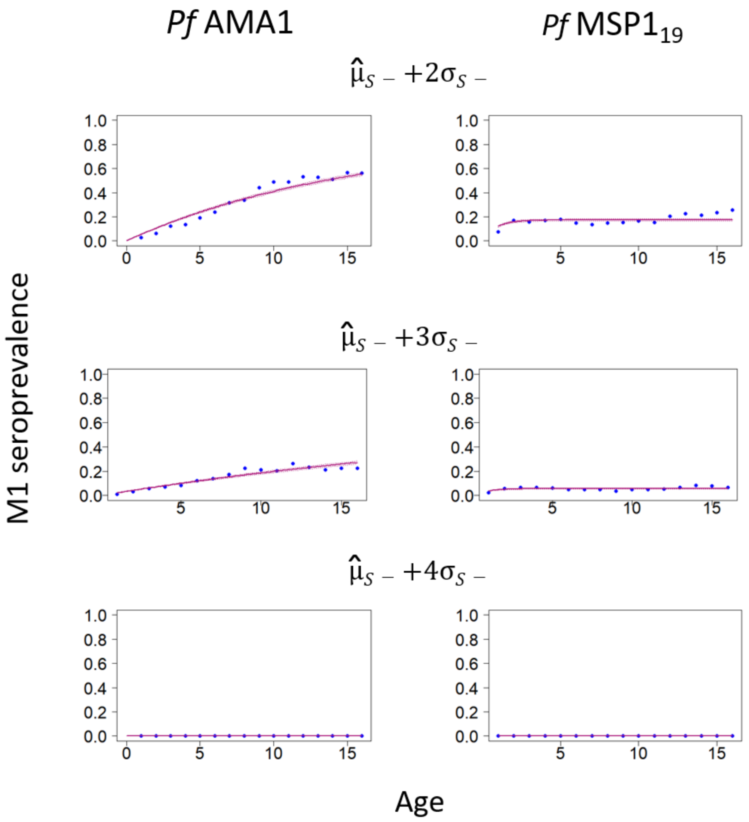

Figure. S 5: The top row shows M1 seroprevalence point estimates (blue dots) where seropositivity is defined as $\mu_{S-} + 2\sigma_{S-}$, as well as the fitted seroprevalence curve (purple curve) and 95% CIs (purple dotted curves) from the RCM. The middle row shows where seropositivity is defined as $\mu_{S-} + 3\sigma_{S-}$, and the bottom row shows where seropositivity is defined as $\mu_{S-} + 4\sigma_{S-}$
